# A new large area MCP-PMT for high energy detection

**DOI:** 10.1038/s41598-023-47818-x

**Published:** 2023-11-22

**Authors:** Lin Chen, Huizhen Yang, Xingchao Wang, Liping Tian, Dongyan Ding, Yunji Wang, Ke Ji, Pengxiang Zheng, Ting Luo, Chenye She

**Affiliations:** 1 School of Network and Communication Engineering, Jinling Institute of Technology, Nanjing, 211169 China; 2 School of Electronic Science and Engineering, Southeast University, Nanjing, 210096 China; 3 North Night Vision Technology (NNVT) CO., LTD, Nanjing, 210110 China

**Keywords:** Electronics, photonics and device physics, Nuclear physics

## Abstract

20-inch Large area photomultiplier tube based on microchannel plate (MCP-PMT) is newly developed in China. It is widely used in high energy detection experiments such as Jiangmen Underground Neutrino Observatory (JUNO), China JinPing underground Laboratory (CJPL) and Large High Altitude Air Shower Observatory (LHAASO). To overcome the poor time performance of the existing MCP-PMT, a new design of large area MCP-PMT is proposed in this paper. Three-dimensional models are developed in CST Studio Suite to validate its feasibility. Effects of the size and bias voltage of the focusing electrodes and MCP configuration on the collection efficiency (CE) and time performance are studied in detail using the finite integral technique and Monte Carlo method. Based on the simulation results, the optimized operating and geometry parameters are chosen. Results show that the mean ratio of photoelectrons landing on the MCP active area is 97.5%. The acceptance fraction of the impinging photoelectrons is close to 100% due to the emission of multiple secondary electrons when hitting the MCP top surface. The mean transit time spread (TTS) of the photoelectrons from the photocathode is 1.48 ns.

## Introduction

20-inch Large area photomultiplier tubes (PMTs) with photon counting capability^1–6^ are widely used in large scale neutrino and cosmic ray experiments. There are generally two types of large area PMTs: existing Dynode-PMTs and newly developed PMTs based on microchannel plate (MCP-PMTs)^6^ which was developed for Jiangmen Underground Neutrino Observatory (JUNO)^7^. JUNO proposed in 2012 is a multipurpose neutrino experiment designed to determine neutrino mass hierarchy and precisely measure oscillation parameters by detecting reactor neutrinos from the Yangjiang and Taishan Nuclear Power Plants, observe supernova neutrinos, study the atmospheric, solar neutrinos and geo-neutrinos, and perform exotic searches. The energy resolution was required to be $3\%\sqrt{E(\mathrm{MeV})}$, which means that the detection efficiency (DE) of the large area photomultiplier tube (PMT) should be above 25%. At that time, DE of R3600, Hamamatsu 20-in. PMT applied in Daya Bay experiment, was just 15.4%. None of the 20-in. Dynode chain PMT product could meet this criterion. In this situation, a 20-in. large area MCP-PMT was proposed and developed by the MCP-PMT collaboration formed by the scientists from Institute of High Energy Physics (IHEP) of the Chinese Academy of Sciences, Northern Night Vision Technology Co., LTD (NNVT) and Xi’an Institute of Optics and Precision Mechanics (XIOPM) of the Chinese Academy of Sciences.

Compared with the typical dynode PMT (R12860 from Hamamatsu)^8^, the MCP-PMT (P6201 from NNVT)^9^ performs well in the rise time, collection efficiency (CE), peak to valley ratio and applied voltage for typical Gain, but in the transit time and transit time spread (TTS), it shows poor performances.

According to the shortcoming of the 20-in. large area MCP-PMT, a good time performance large area MCP-PMT design accompanied with high CE is proposed in this work. Simulations are conducted to systematically investigate CE and time performance for various operating and geometry parameters including the size and bias voltage of the two focusing electrodes and MCP. Based on the simulation results, a set of operating and geometry parameters are chosen.

## Concept design and simulation

**Figure 1:**
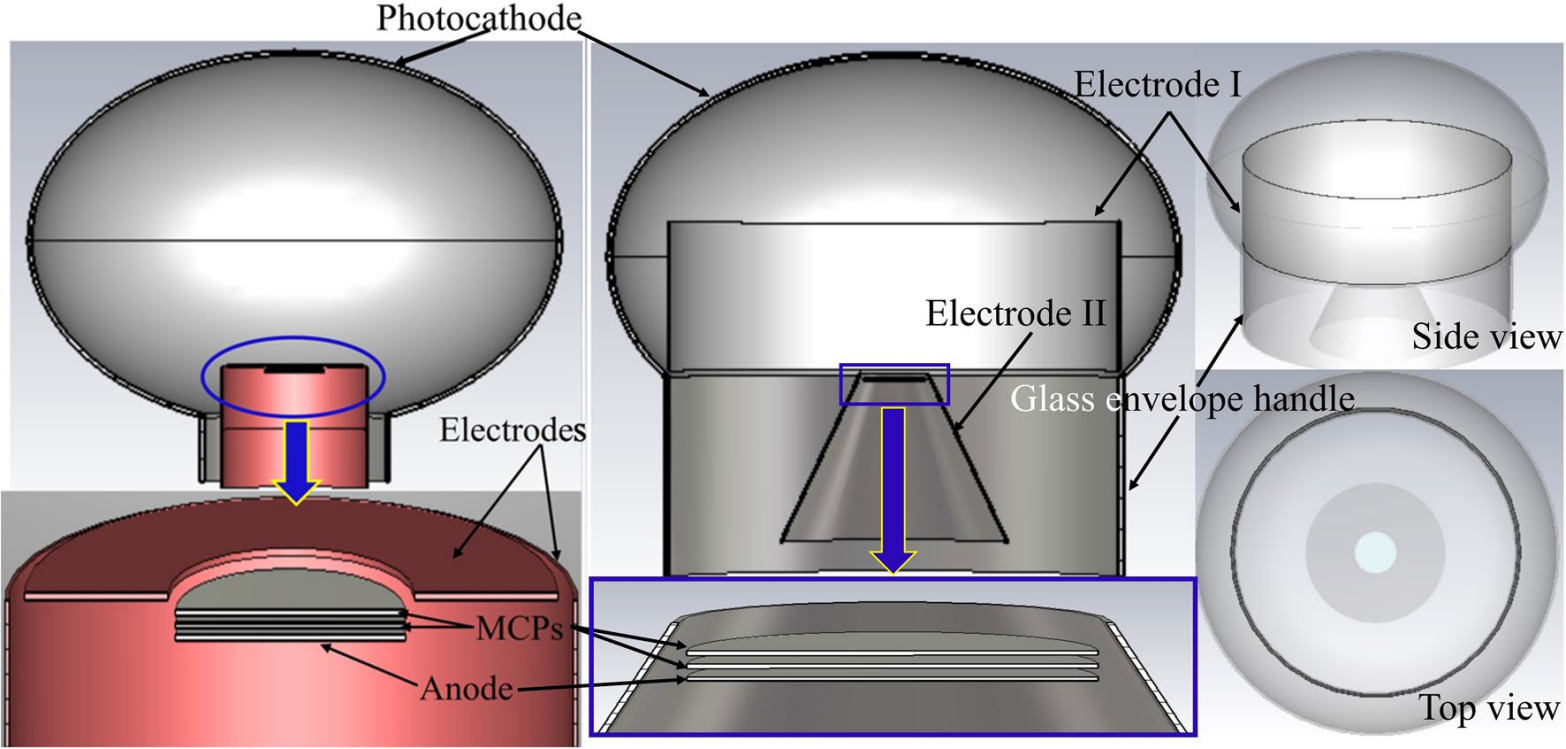
Schematic diagrams of the existing (left side) and new (right side) large area MCP-PMTs.

To obtain a good time performance, a new large area MCP-PMT with Ф 460 mm photocathode diameter is proposed in this paper. The schematic diagram is exhibited in Fig. 1 (right side). Compared to the existing MCP-PMT (left side), the structure of the new design has been greatly changed. Generally, in the PMT cavity, the curved equipotential surface shows better focusing characteristic, and the planar equipotential surface benefits the consistency of the transit time. To obtain both high CE and short TTS, the focusing system of the new design includes a cylindrical barrel (Electrode I, designed for short TTS) and a conical barrel (Electrode II, designed for high CE). A pair of MCPs are placed at the top opening of the conical barrel. Implementing millions of MCP channels in the three-dimensional model is impossible. Hence, a simplified model based on two MCPs without channels is adopted in our simulation. The glass envelope handle cooperates with the Electrode I, Electrode II and MCPs to generate the accelerating and focusing electric field which benefits CE and time performances.

Simulations are conducted to validate the feasibility. The new MCP-PMT model is simulated in CST Studio Suite^10^. The electric field, electron trajectories, energies and velocities are calculated based on the Finite Integral Technique and Monte Carlo method. The feasibility and effectiveness of this simulation approach has already been validated^11–13^.

Photoelectron trajectories from the photocathode to MCP are well simulated. 2000 photoelectrons uniformly distributed from 28.5° (corresponding to the photocathode edge) to 90°on photocathode are sampled by Monte Carlo method. The initial energy of electrons exiting the photocathode obeys β(1,4) distribution with mean value 0.15 eV in the range 0.0–0.6 eV. The emitted azimuth is uniform distributed over the range of 0–2π. Initial elevation follows Lambert cosine distribution from 0° to 90°. Owing to the short distance and high potential difference between the first MCP-in and anode, the electron transit time through the MCP is around several hundred picoseconds and TTS is just tens of picoseconds which thus are negligible. In our simulation, the transit time distribution between the photocathode and the first MCP is evaluated.

CE is defined as a ratio of the number of photoelectrons from the photocathode collected by MCP channels to the total number. Large area MCP-PMT employs coated MCP which is coated with high secondary electron yield (SEY) material on the flat inter-channel area and electrode penetrating in channels area, to obtain high CE^11,14^. A great deal of secondaries can be excited in the high SEY area and finally be collected by the MCP channels, which makes the acceptance fraction of the impinging photoelectrons close to 100%^11^. In our simulation, it is impossible to obtain the exact CE value attributing to the simplified MCP model approximated to a flat surface without channels. Thus, only the ratio of photoelectrons landing on the simplified MCP input flat surface (active area, CE_a_) is evaluated.

The dependence of CE_a_ and time performance on the bias voltage and size of the two focusing electrodes and MCP is systematically investigated. Photocathode voltage is 0 V. Only one parameter is varied at a time, while the others are kept constant, with values listed in Table 1.

**Table 1:** Fixed parameters of the new MCP-PMT.

Item	Parameter	Value
Glass envelope	Major axis /mm	ϕ 508
	Minor axis /mm	ϕ 360
	Handle diameter (D_h_)/mm	ϕ 400
Electrode I	Diameter (D_I_) /mm	ϕ 400
	Hight (H_I_) /mm	130
	Bias voltage (U_I_) /V	100
Electrode II	Bottom diameter (D_II-b_) /mm	ϕ 200
	Top diameter (D_II-t_) /mm	ϕ 60
	Hight (H_II_) /mm	150
	Bias voltage (U_II_) /V	2000
MCP	Bias voltage (U_MCP_) /V	2500
Photocathode	Diameter /mm	ϕ 460
	Bias voltage /V	0

## Simulation results and discussions

### Electrode I

**Figure 2:**
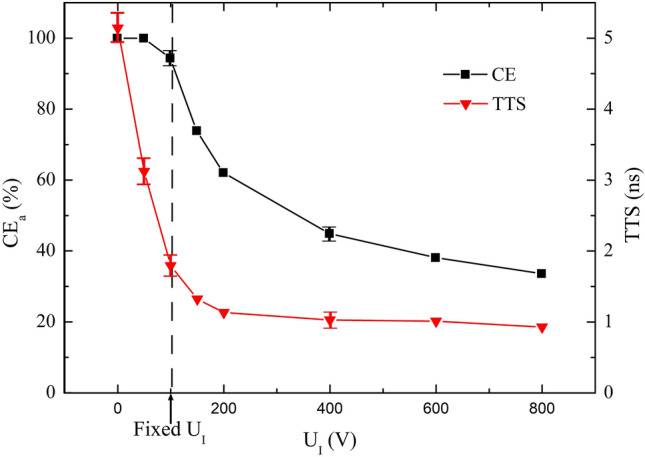
CE_a_ and TTS versus U_I_ over the range of 0 V ≤ U_I_ ≤ 800 V.

The dependence of CE_a_ and time performance on the applied voltage (U_I_), diameter (D_I_) and height (H_I_) of the electrode I is investigated. U_I_, H_I_ and D_I_ are varied from 0 to 800 V, 20 mm to 170 mm and 240 mm to 400 mm, respectively. For each computing, only one parameter is varied at a time, while the others are kept constant, with values listed in Table 1. Sample results with values of mean ± SD are graphically represented in Figs. 2, 4 and 5.

**Figure 3:**
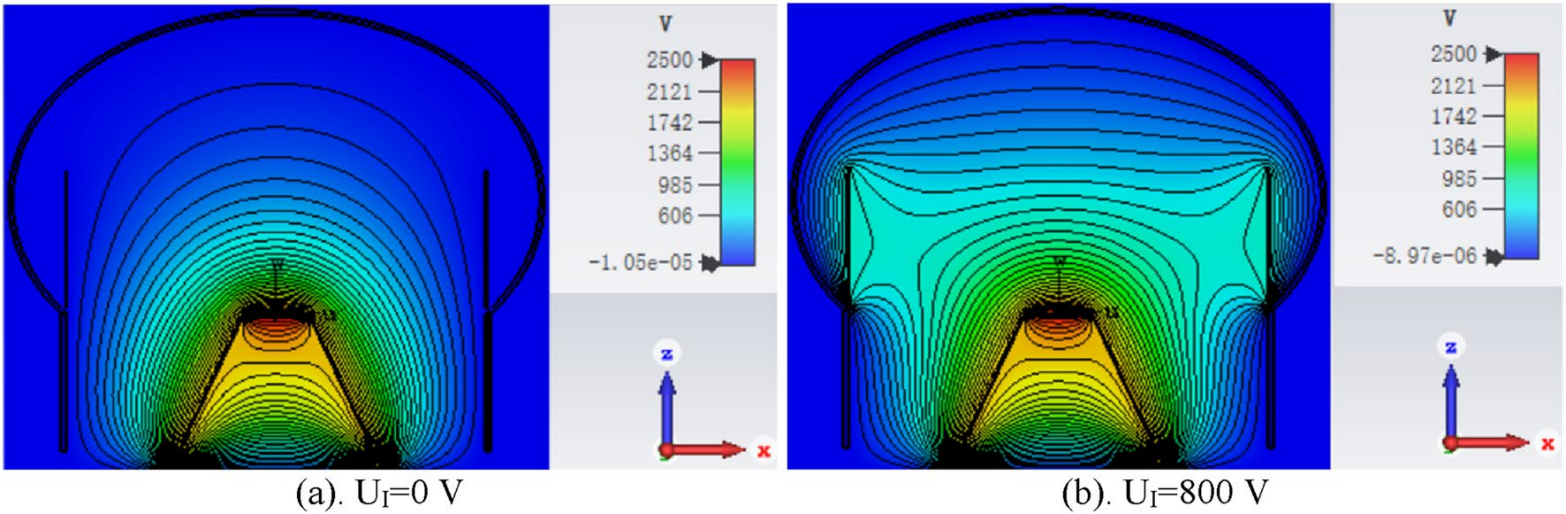
Electric field distributions in the PMT cavity for U_I_ = 0 V (**a**) and 800 V (**b**).

In Fig. 2, decreasing CE_a_ and TTS are observed as results of increasing U_I_. Figure 3 shows the electric field distributions in the PMT cavity for U_I_ = 0 V (a) and 800 V (b). For U_I_ = 0 V the electric field shows better focusing characteristic. It is helpful to obtain a high CE_a_. For U_I_ = 800 V, the electric field between the photocathode and the top opening of electrode I is more uniform, which benefits the consistency of transit time which means short TTS. The CE_a_ at U_I_ = 0 V and 50 V is 100%. The shortest mean TTS is 0.93 ns at U_I_ = 800 V.

**Figure 4:**
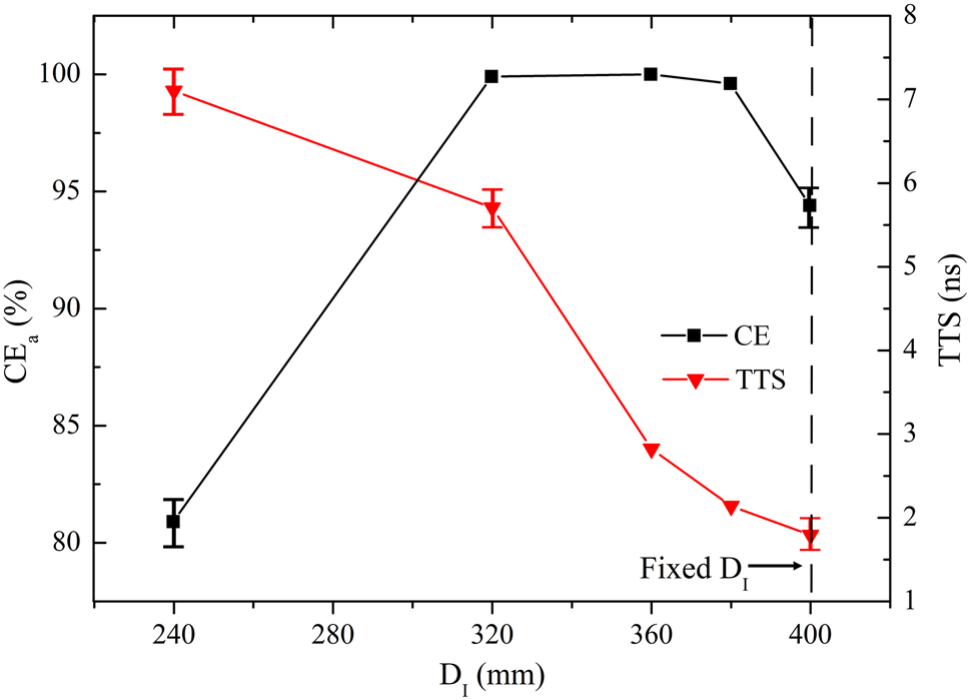
CE_a_ and TTS versus D_I_ over the range of 240 mm ≤ D_I_ ≤ 400 mm.

It is obvious in Fig. 4 that CE_a_ gradually increases to a maximum of 100% at D_I_ = 360 mm and then reduces to some extent with increasing D_I_. Besides, a decreased TTS by increasing D_I_ is observed. The shortest mean TTS is 1.79 ns at D_I_ = 400 mm. For smaller D_I_, the electric field shows better focusing property. Larger D_I_ is benefit for the uniformity of electric field, and finally TTS.

**Figure 5:**
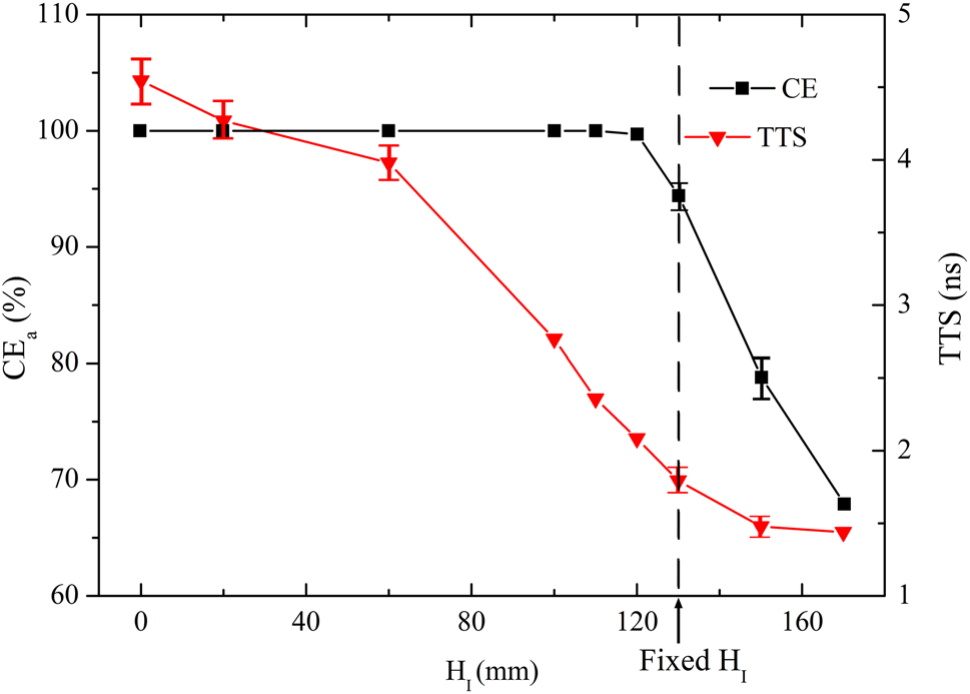
CE_a_ and TTS versus H_I_ over the range of 0 mm ≤ H_I_ ≤ 170 mm.

Figure 5 exhibits the simulated CE_a_ and TTS, which result from the variation of H_I_ over the range of 0–170 mm. CE_a_ and TTS basically decrease with the increasing H_I_. From the visual electric distribution in the PMT cavity, it is observed that the electric field for lower H_I_ shows better focusing property. Higher H_I_ is benefit for the electric field uniformity. CE_a_ is 100% for 0 mm ≤ H_I_ ≤ 110 mm. The shortest mean TTS is 1.44 ns at H_I_ = 170 mm.

### Electrode II

**Figure 6:**
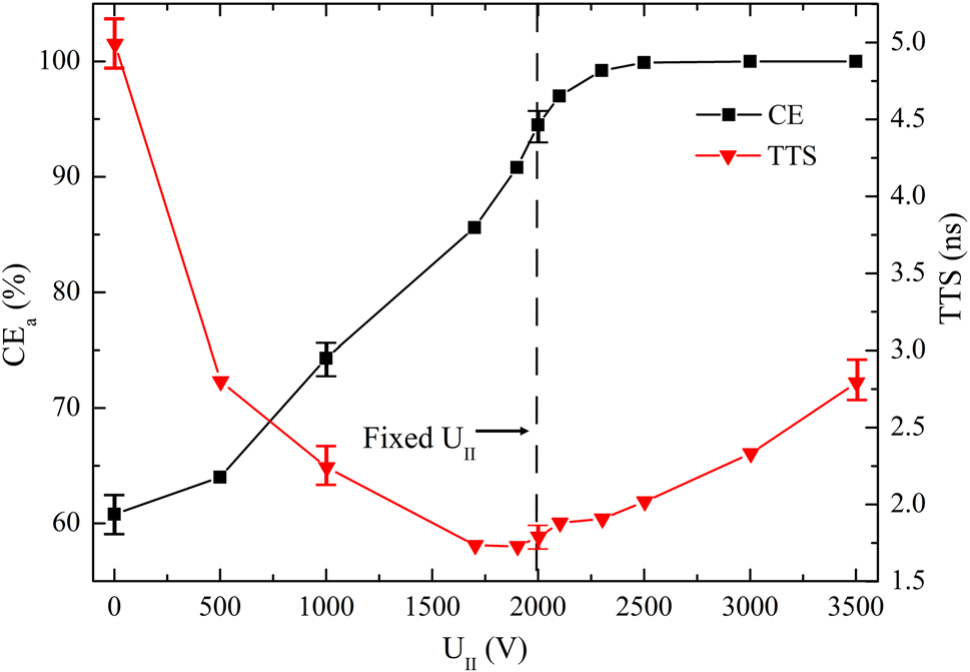
CE_a_ and TTS versus U_II_ over the range of 0 V ≤ U_II_ ≤ 3500 V.

**Figure 7:**
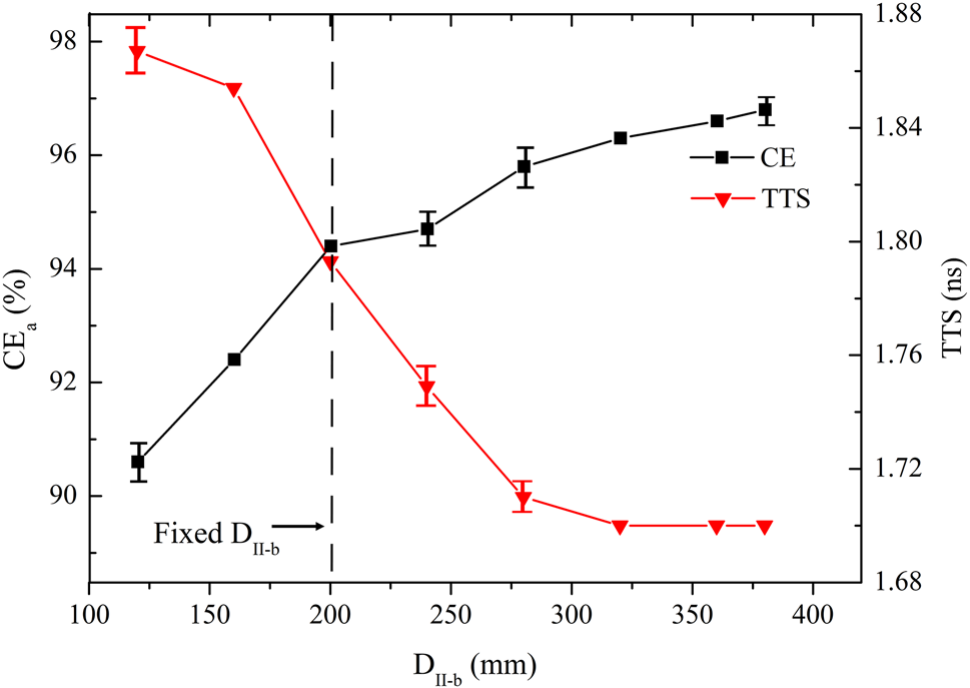
CE_a_ and TTS versus D_II-b_ over the range of 120 mm ≤ D_II-b_ ≤ 380 mm.

**Figure 8:**
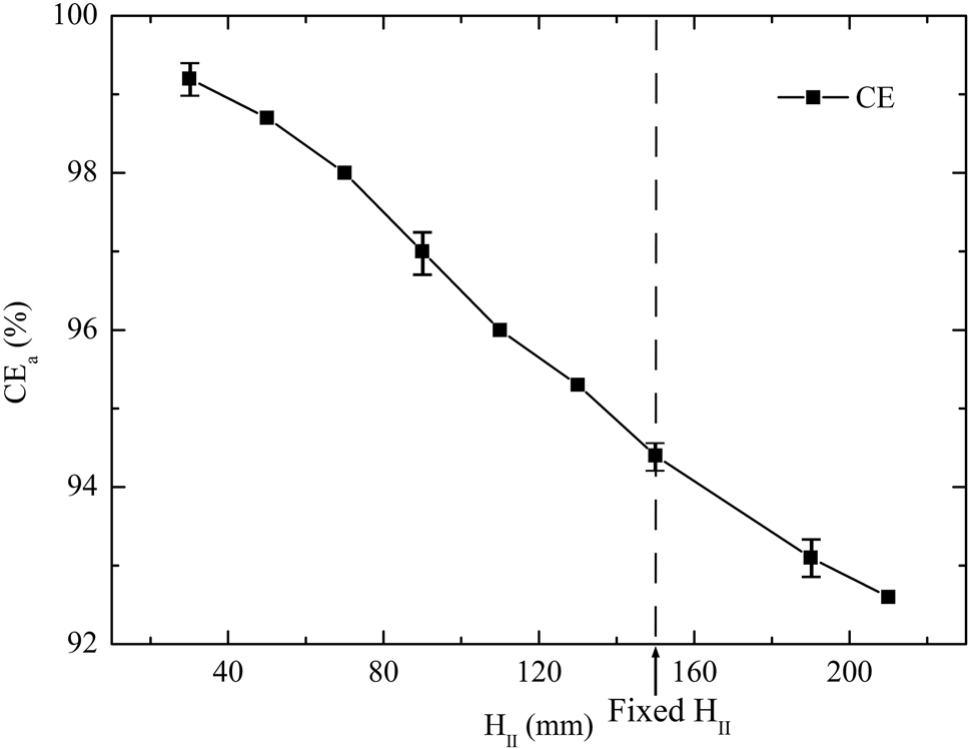
CE_a_ versus H_II_ over the range of 30 mm ≤ H_II_ ≤ 210 mm.

The applied voltage (U_II_), bottom diameter (D_II-b_) and height (H_II_) of the electrode II have significant impacts on CE_a_ and time performance. U_II_, H_II_ and D_II-b_ are varied from 0 to 3500 V, 120–380 mm and 30–210 mm, respectively. For each computing, only one parameter is varied at a time, while the others are kept constant, with values listed in Table 1. Sample results with values of mean ± SD are graphically represented in Figs. 6, 7 and 8.

As can be seen in Fig. 6 that with the increasing U_II_, CE_a_ increases to 100% at U_II_ = 3300 V, and the mean TTS decreases until its minimum 1.73 ns at U_II_ = 1900 V and then increases. With the increasement of U_II_, the electric field is divergent first, then approximately uniform (benefit for short TTS), finally focused (conducive to high CE_a_).

It is shown in Fig. 7 that increasing D_II-b_ has positive effects on CE_a_ and TTS. Electric field for smaller D_II-b_ shows better focusing property. Larger D_II-b_ is benefit for the field uniformity. D_II-b_ = 380 mm is the optimum value, for which the mean CE = 96.8% and mean TTS = 1.7 ns.

Increasing H_II_ has a slight negative effect on CE_a_ (exhibited in Fig. 8) but no significant effect on TTS. The mean TTS changes from 1.74 to 1.85 ns over the range of 30 mm ≤ H_II_ ≤ 210 mm. At H_II_ (fixed value) = 150 mm, mean TTS is 1.79 ns. The highest mean CE_a_ is 99.2% at H_II_ = 30 mm.

### MCP

**Figure 9:**
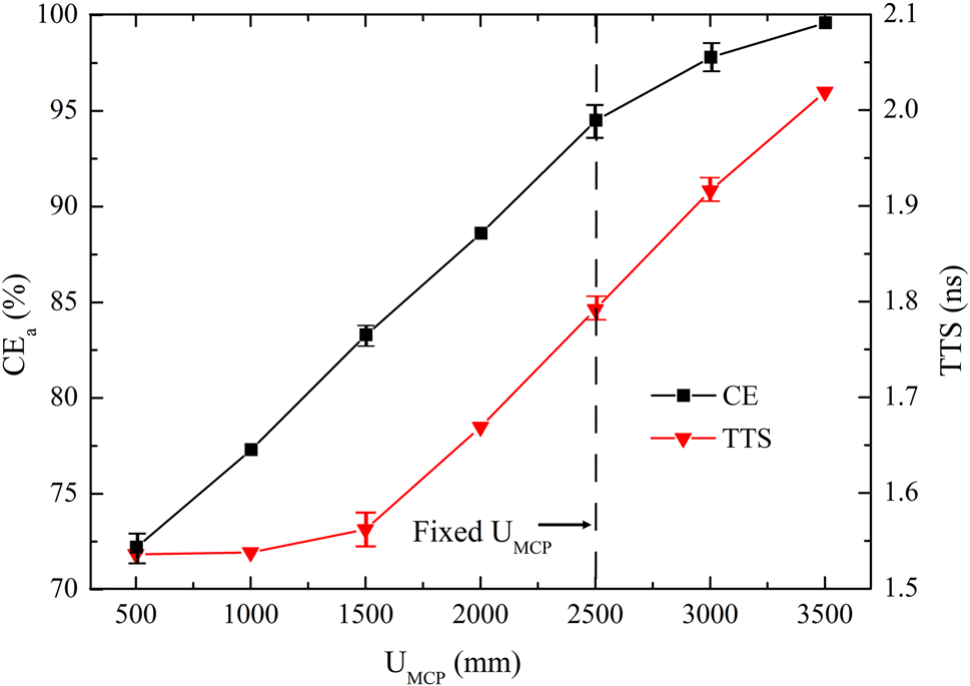
CE_a_ versus U_MCP_ over the range of 500 V ≤ U_MCP_ ≤ 3500 V.

Dependence of CE_a_ and TTS on the input face of the top MCP applied voltage (U_MCP_) is well simulated. The total voltage applied on the two MCPs is 1000 V. Only U_MCP_ is varied, while the others are kept constant, with values listed in Table 1. As is shown in Fig. 9 that with the increase of U_MCP_, CE_a_ and TTS increase. Electric field for higher U_MCP_ shows better focusing property but poorer uniformity. The highest mean CE_a_ is 99.6% at U_MCP_ = 3500 V. The shortest mean TTS is 1.54 ns at U_MCP_ = 500 V.

### Optimized model

Considering both high CE_a_ and good time performance requirements, U_I_ = 100 V, D_I_ = 400 mm, H_I_ = 130 mm, U_II_ = 2000 V, D_II-b_ = 380 mm, H_II_ = 30 mm and U_MCP_ = 2500 V are employed in the MCP-PMT model. Other parameter values are listed in Table 1. Simulation results show that the new design has a high CE and outstanding time performance.

**Figure 10:**
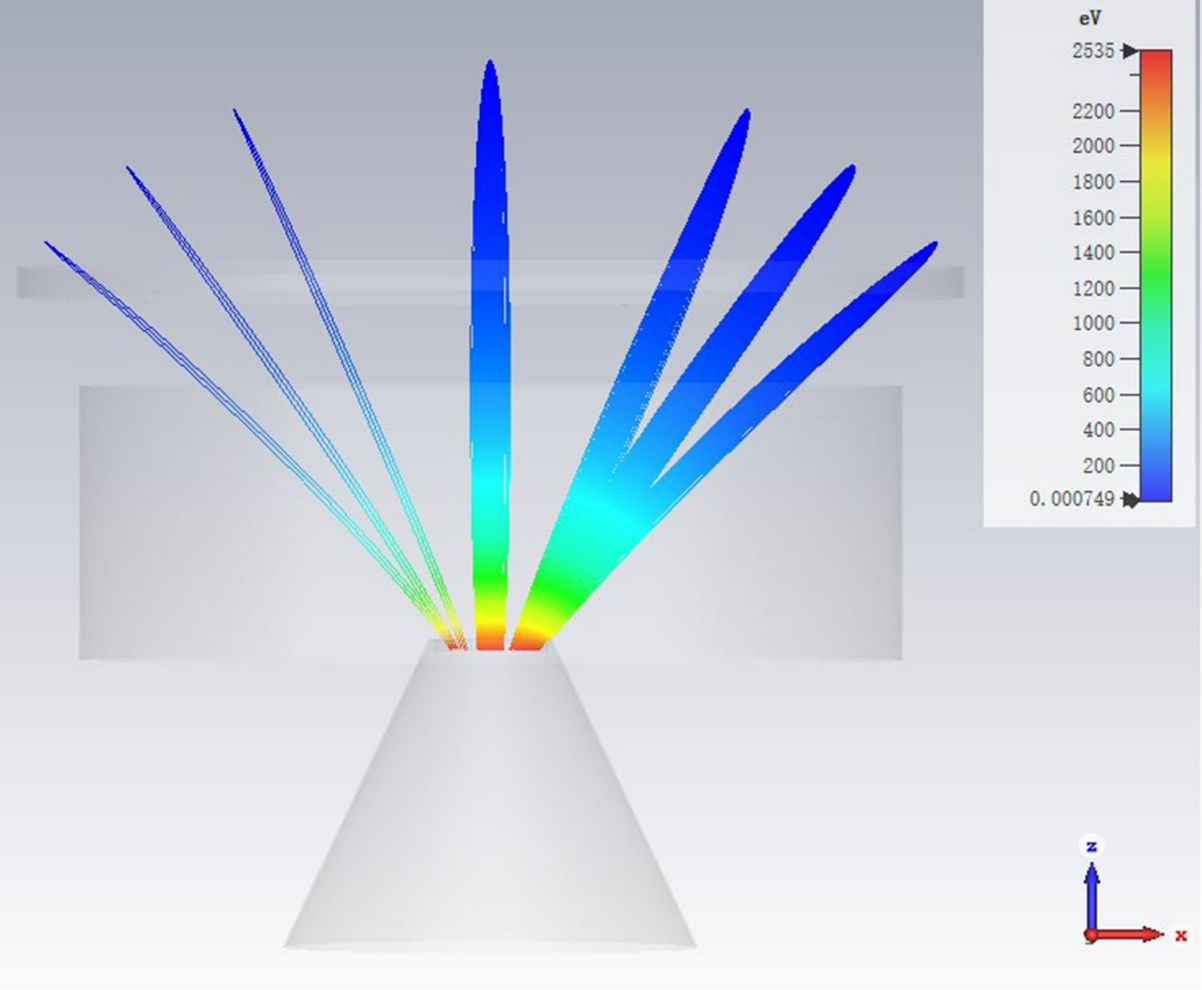
Electron trajectories from the photocathode to the MCP input face.

Electron trajectories from the photocathode to the MCP input face is shown in Fig. 10. On the right side, the photoelectron trajectories from four points on the latitudes 30°, 45°, 60° and 90° with the mentioned initial angle spread are exhibited, and on the left side, photoelectron trajectories for such positions with initial angles of 30°, 45° and 60° (corresponding to the X–Z coordinates) are shown.

**Figure 11:**
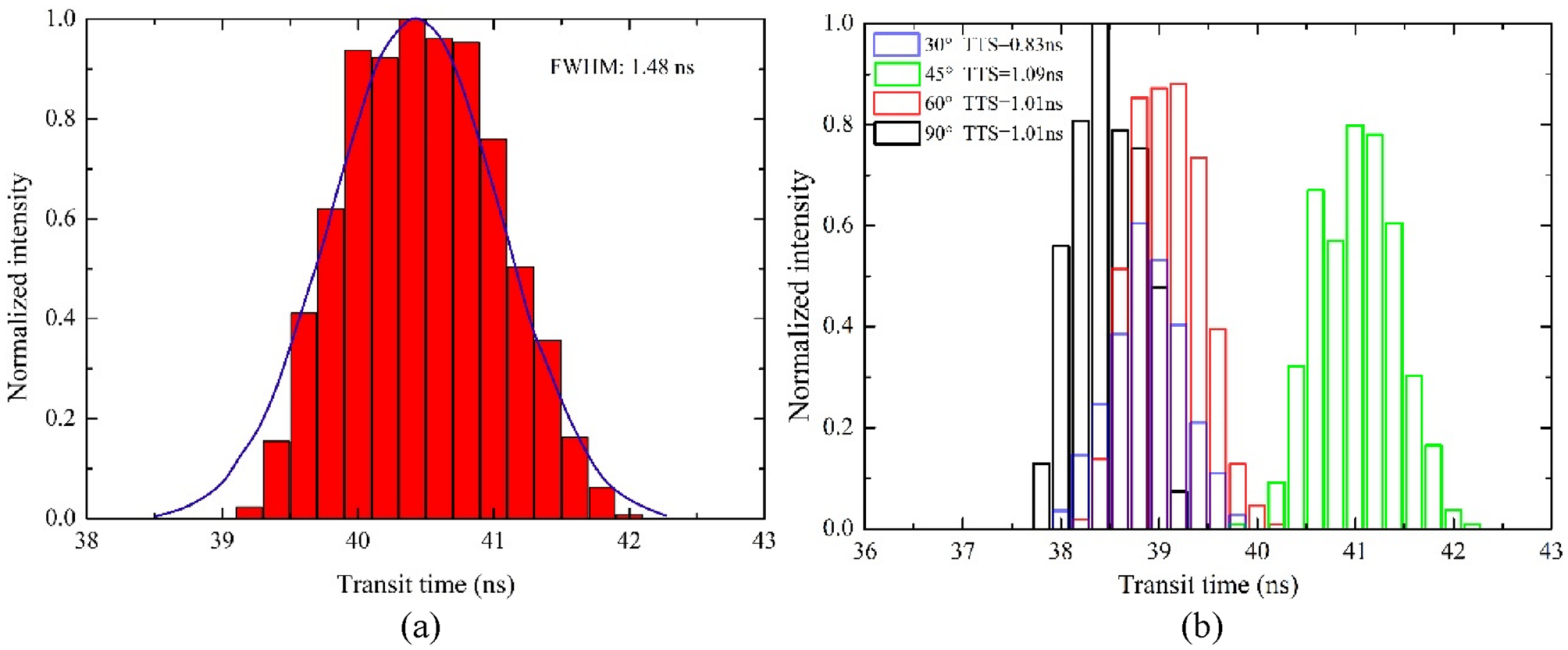
Photoelectron transit time distributions. (**a**). Photoelectrons emitted from the whole photocathode. (**b**). Photoelectrons emitted from the latitudes 30°, 45°, 60° and 90° of the photocathode, respectively. FWHM is TTS.

A sample transit time distribution result of 2000 photoelectrons from the whole photocathode is exhibited in Fig. 11a. The transit time and TTS are 40.5 and 1.48 ns, which are greatly improved than 120 and 15 ns of the existing MCP-PMT^9^ and 95 and 2.4 ns of the dynode PMT^8^. Transit time distributions of photoelectrons from latitudes 30°, 45°, 60° and 90° are shown in Fig. 11(b). Affected by the electric field in the PMT cavity, latitude 45° corresponds to the largest TTS which is 1.09 ns. Besides, results shows that the mean TTS is also 1.48 ns.

**Figure 12:**
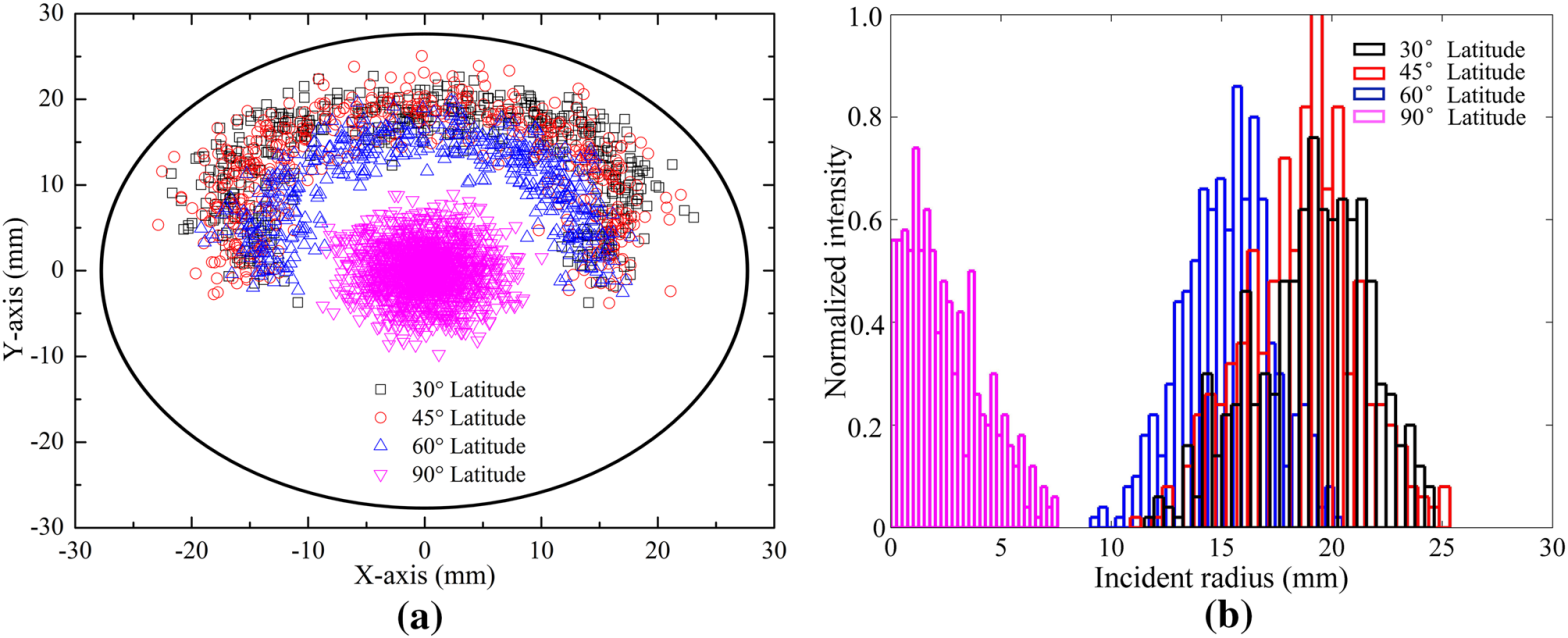
Incident photoelectron distribution on the MCP input face. 90° Latitude corresponding to the top point. Considering the symmetry of the MCP-PMT, photoelectrons are emitted from half of the photocathode. (**a**). Incident photoelectron position distribution on the MCP input face. (**b**). Incident radius distribution on the MCP input face.

Photoelectrons incident positions on the MCP input face are well evaluated. Results are shown in Fig. 12. Photoelectrons from different latitudes land at different diameters on the MCP input face. With the increasing latitude, this effect become more obvious. For 30° and 45° Latitudes, the incident positions are indistinguishable. Simulation results show that the mean CE_a_ of the photoelectrons from the whole photocathode is 97.5%, which mean that CE of the coated MCP-PMT is expected to be 100%.

## Conclusion

This work presents a new large area MCP-PMT design with good time performance. A novel focusing system with a cylindrical barrel electrode and a conical barrel electrode are designed. Three-dimensional models are developed in CST Studio Suite to validate the feasibility and effectiveness. Dependences of CE and time performance on the size and bias voltage of the focusing electrodes and MCP configuration are systematically investigated. Based on the simulation results, a set of operating and geometry parameters are chosen for the new MCP-PMT design considering both high CE and good time performance. Results show that the mean ratio of photoelectrons landing on the channelless MCP input flat surface is 97.5%. The mean TTS value of photoelectrons from the whole photocathode achieves 1.48 ns. It will be a good candidate for the detection experiments with high CE and high time resolution requirements.

## Data Availability

The datasets used and/or analysed during the current study available from the corresponding author on reasonable request.
